# Assessing sun protection practices for children: knowledge and behaviours of parents: Diana Nemes

**DOI:** 10.1093/eurpub/ckac131.361

**Published:** 2022-10-25

**Authors:** D Nemes, MA Coman

**Affiliations:** 1 Department of Public Health, Babes-Bolyai University, Cluj-Napoca, Romania; 2 Center for Health Workforce Research and Policy, Babes-Bolyai University, Cluj-Napoca, Romania

## Abstract

**Background:**

The development of melanoma in adulthood is strongly associated with sunburns during childhood. Parental knowledge and behaviours play a key role in sun protection behaviour from which children can acquire general and integrated learning patterns. With this being known, numerous positive preventive health behaviours can be initially shaped in the family, with children having parents as a model.

**Methods:**

A cross-sectional approach (web-based questionnaire) was conducted to gather information regarding parents’ knowledge and behaviours of children’s sun protection, alongside the predictors that might influence the adoption of these behaviours, between April and May 2021. The survey was disseminated to 53 primary school teachers from 9 schools in Cluj-Napoca Romania, and the data set included 355 valid surveys (parents with at least one child aged between 0 to 12 years old) out of 476 total surveys. Descriptive statistics, Chi-square tests of association and logistic regressions were computed.

**Results:**

The study showed differences in children’s sun exposure patterns, their sunburn and parental sun protection behaviour. Overall, parents reported fair sun protection behaviours and children’s sunburn frequency was overall moderate among all children in the previous summer season. However, an increase in children’s age generates an increase in parents’ sunscreen application for their children in both planned and incidental situations. There were statistically significant associations between parents’ sex and their knowledge about the fatal consequences of skin cancer or their level of education and the damage produced by tanning bed usage or sunscreen efficiency measures.

**Conclusions:**

These results are a starting point for various program interventions that can be done for parents in order to increase their knowledge on sun protection practices for their children.

**Key messages:**

• Skin cancer is substantially preventable if unprotected exposure to ultraviolet radiation is reduced during the first years of a child’s life.

• Parental knowledge and behaviors play a key role in sun protection behavior from whom children can acquire general and integrated learning patterns.

